# Kinect-based assessment of proximal arm non-use after a stroke

**DOI:** 10.1186/s12984-018-0451-2

**Published:** 2018-11-14

**Authors:** K. K. A. Bakhti, I. Laffont, M. Muthalib, J. Froger, D. Mottet

**Affiliations:** 1 Euromov, University of Montpellier, Montpellier, France; 2 Physical Medicine and Rehabilitation, Montpellier University Hospital, Montpellier, France; 3 Silverline Research, Brisbane, Australia; 4 Physical Medicine and Rehabilitation, Nîmes University Hospital, Le Grau du Roi, France; 5 Federative Institute for Research on Handicap, Paris, France

**Keywords:** Arm non-use, Stroke, Rehabilitation, Kinect v2, Movement analysis

## Abstract

**Background:**

After a stroke, during seated reaching with their paretic upper limb, many patients spontaneously replace the use of their arm by trunk compensation movements, even though they are able to use their arm when forced to do so. We previously quantified this proximal arm non-use (PANU) with a motion capture system (Zebris, CMS20s). The aim of this study was to validate a low-cost Microsoft Kinect-based system against the CMS20s reference system to diagnose PANU.

**Methods:**

In 19 hemiparetic stroke individuals, the PANU score, reach length, trunk length, and proximal arm use (PAU) were measured during seated reaching simultaneously by the Kinect (v2) and the CMS20s over two testing sessions separated by two hours.

**Results:**

Intraclass correlation coefficients (ICC) and linear regression analysis showed that the PANU score (ICC = 0.96, r^2^ = 0.92), reach length (ICC = 0.81, r^2^ = 0.68), trunk length (ICC = 0.97, r^2^ = 0.94) and PAU (ICC = 0.97, r^2^ = 0.94) measured using the Kinect were strongly related to those measured using the CMS20s. The PANU scores showed good test-retest reliability for both the Kinect (ICC = 0.76) and CMS20s (ICC = 0.72). Bland and Altman plots showed slightly reduced PANU scores in the re-test session for both systems (Kinect: − 4.25 ± 6.76; CMS20s: − 4.71 ± 7.88), which suggests a practice effect.

**Conclusion:**

We showed that the Kinect could accurately and reliably assess PANU, reach length, trunk length and PAU during seated reaching in post stroke individuals. We conclude that the Kinect can offer a low-cost and widely available solution to clinically assess PANU for individualised rehabilitation and to monitor the progress of paretic arm recovery.

**Trial registration:**

The study was approved by The Ethics Committee of Montpellier, France (N°ID-RCB: 2014-A00395–42) and registered in Clinical Trial (N° NCT02326688, Registered on 15 December 2014, https://clinicaltrials.gov/ct2/show/results/NCT02326688).

## Background

A large proportion of post-stroke individuals develop upper limb (UL) disabilities that result in difficulties in performing daily activities such as reaching for objects, which impacts their quality of life [1]. Stroke survivors often compensate for these impairments by adapting their movement patterns to incorporate additional degrees of freedom at new joints and body segments. One of the most common compensatory behaviours following a stroke is when patients replace the use of the paretic UL with the use of the less impaired UL. When such compensation persists even if the paretic UL recovered enough to be used, this is called learned non-use. However, the learned non-use phenomenon is more general than this specific case: it applies to any cases when individuals “forget” how to use their affected body parts and instead overuse compensations [2]. Here, our reasoning is that the learned non-use phenomenon explains why some patients “forget” how to use the proximal joints of their paretic UL and instead overuse trunk compensation during reaching [3]. Long-term non-use of shoulder-elbow movements is suspected to be detrimental to optimal upper limb functional recovery, due the “Use it or lose it” principle of use-dependent neural plasticity [4, 5]. Long-term overuse of trunk compensation may lead to suboptimal motor recovery of the paretic UL and secondary complications such as muscle contractures [6–8].

In our previous study [3], the non-use of shoulder-elbow joints during reaching has been termed “proximal arm non-use” and was assessed with the PANU score. The PANU score was derived by the substraction of the spontaneous proximal arm-use (SPAU) from the maximal proximal arm-use (MPAU). MPAU was recorded when patients were asked to voluntarily maximise proximal arm-use (or constrained not to use trunk movement). SPAU was recorded when patients were free to spontaneously balance their use of trunk and their use of proximal arm joints during hand reaching while seated. We showed that the PANU score had very good test-retest reliability. The PANU score did not depend on time since stroke. Higher PANU scores were moderately related to higher UL impairment (Fugl Meyer Assessment of the upper extremity-FM-UE) and to lower UL function (Box & Block Test-BBT). Moreover, 61% of patients with lower impairment (FM-UE proximal > 28/42) exhibited proximal arm non-use (PANU score > 6.5%) [3]. The PANU score provides information about the remaining functional motor reserve of the paretic arm (at the level of shoulder and elbow movements), which is not used by the post-stroke individual. Consequently, the PANU score potentially enables a clinician to select patients for specific rehabilitation programs focusing on the “maximal-use” of shoulder and elbow movements as well as to monitor the paretic arm recovery/compensation post-stroke. Therefore, PANU assessment is complementary with routine clinical measures of arm impairment (e.g., FM-UE) and function (e.g., BBT).

In our previous study [3], the PANU measurement using the ultrasound 3D motion capture system (CMS20s, Zebris) required to position markers on the hands and on the trunk of the patient during the seated reaching task. Although most clinical motion analysis devices use a marker-based system [9], recent developments in computer gaming technology brought forward marker-less infrared sensors such as the Microsoft’s Kinect (v2 for Xbox One) that captures the users’ body movement and allows them to interact within video games [10–14]. The Kinect sensor is low cost and reliably tracks body joints in real-time without requiring markers attached to the body [15]. Moreover, the official Microsoft software development kit (SDK) release permitted the Kinect sensor to be used not only as a gaming device, but also as a measurement system. The Kinect sensor showed comparable results in measuring shoulder movements during validity assessment with an established highly accurate 3D motion capture (6-camera Vicon) system [16, 17]. However, very few studies are available on the validity of the Kinect to accurately track trunk and hand motions in a reaching task [18], which is essential for quantifying the PANU score in post-stroke individuals.

Therefore, the purpose of this study was to validate a Kinect-based system to accurately and reliably quantify PANU scores and related kinematic parameters in individuals who sustained a stroke. Our clinical question was whether the Kinect sensor could replace the existing CMS20s sensor for PANU assessment. We hypothesised that the Kinect will provide similar measurements of PANU score and related kinematic variables as those determined by the reference CMS20s system.

## Methods

### Participants

**Fig. 1:**
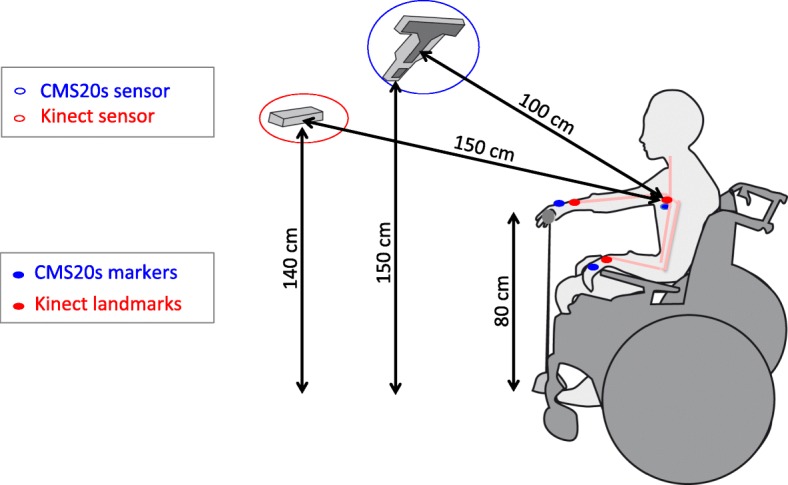
Experimental setup. The quantification of the proximal arm non-use (PANU) score was simultaneously determined by the Kinect (blue encircled) and CMS20s (red encircled) movement recording systems. The CMS20s recorded the position of 3 markers placed on the manubrium, right dorsal hand, left dorsal hand (blue spots). The Kinect provided a skeleton of the person (orange) out of which we retained 3 “joints” corresponding best to the position of CMS20s markers on the body: Spine-Shoulder, WristRight, WristLeft

A total of 19 people who had suffered a stroke (59 ± 3 years; 9 women) participated in this study. Participants were recruited during their in-patient hospitalization at the department of Physical and Rehabilitation Medicine of Nimes University Hospital Le Grau du Roi and Montpellier University Hospital Lapeyronie. We chose patients with no restriction of time since their stroke, which resulted in a wide range from 13 to 4027 days after stroke with a median of 96 days after a stroke. Inclusion criteria were: single supratentorial cerebral vascular accident; either haemorrhagic or ischemic; any time after the stroke; either left or right affected hemisphere; aged ≥18 and ≤ 90; able to carry out a seated hand reaching task with the paretic arm (see details of reaching task in Fig. 1). Participants were excluded if they had shoulder pain or perceptual-cognitive deficits (hemi-negligence, ataxia, receptive aphasia) [19]. The study was approved by The Ethics Committee of Montpellier, France (N°ID-RCB: 2014-A00395–42) and registered in Clinical Trials (N° NCT02326688). Written informed consent was obtained from all participants prior to their inclusion.

### Clinical assessments

The patient’s UL sensorimotor impairment was assessed with the FM-UE, allowing a maximal score of 66 [20]. The patient’s UL functional capacity was measured using the BBT, with scores expressed as the number of blocks transferred using the paretic arm [21]. These two clinical tests were used in our previous study [3].

### Experimental protocol

The participants seated comfortably on a chair with armrests and were invited to reach a target placed in front of them, with each of their hands. The start position was standardised with the pronated forearm resting on the armrest such that the hand was hanging without support. The target was aligned to the midline of the body at a distance determined in terms of the length of the participant’s active stretched arm measured from the medial axilla to the distal wrist crease [3, 22]. The reaching movement speed was self-selected and was repeated 5 times with the paretic hand, then 5 times with the less-impaired hand. This reaching sequence was first performed in a spontaneous proximal arm-use condition (SPAU), and then in a maximal proximal arm-use condition (MPAU). In the SPAU condition, after a “go” signal, the participant was free of any constraint and reached the target in a spontaneous manner. In the MPAU condition, the therapist provided manual (Therapists’ hands on the patient’s sternum) and verbal feedback to ensure that the patient minimized trunk use as much as possible and, consequently, maximised the use of the elbow-shoulder joints [3, 23, 24]. The sequence of reaching tasks to assess the PANU score was repeated 2 h later to determine test-retest reliability and to know the magnitude of the difference in PANU score due to the patient repeating the tasks (i.e., practice effect). The same therapist evaluated each participant in the two assessments.

### Experimental setup

The experimental setup of the Kinect and CMS20s sensors allowed simultaneous capturing of the 3D motion of the trunk and hand during seated reaching by the two systems (see details in Fig. 1).

#### CMS20s sensor

The CMS20s (Zebris, Isny, Germany) is a marker-based system that measures body kinematics based on travel-time measurement of ultrasound impulses. Ultrasound impulses emitted by miniature markers placed on the body are triangulated by three microphones built into the CMS20s receiver at a sampling rate of 50 Hz. The CMS20s was chosen as the gold standard motion analysis system (accuracy lower than 1/10 mm). Three markers were placed so as to be clearly visible by the CMS20s during the whole reach sequence: on the right and left head of the second metacarpal (dorsal hand) and on the manubrium (blue spots in Fig. 1). The CMS20s system was connected to a PC running the “WinData” software (Zebris, Isny, Germany) to record the position time-series of the markers.

#### Kinect sensor

The Kinect (v2, Microsoft, USA) is a marker-less motion capture system combining 3 sensors (a RGB colour camera, a depth sensor, an infrared sensor) to provide the 3D position (X, Y, Z) of 25 landmarks on a skeleton with a sampling rate of 30 Hz [25]. The Kinect processes the distance data by a time-of-flight camera system with proprietary algorithms. The time-of-flight system is able to reconstruct the 3D scene through the measurement of the time elapsed between the emission of a light ray and its collection after reflection on the target [26]. The Kinect was connected to a PC running the “MaCoKi” software (NaturalPad, Montpellier, France) developed from the Kinect SDK (v2.0_1409, Microsoft, USA) to record the position time-series of the hands and trunk.

#### Position of landmarks for the CMS20s and for the Kinect

The Kinect records the movements of 25 predefined body “joints” that approximately correspond to the centre of the anatomical joint or body part. For the comparison of the position of landmarks between the two systems, we had to select the Kinect joints best corresponding to the CMS20s markers. Preliminary tests revealed that the “wrist” in the Kinect-based records corresponded best to the positioning of the CMS20s marker on the dorsal face of the hand. Similarly, “Spine-Shoulder” in the Kinect-based records corresponded best to a marker on the manubrium (red spots in Fig. 1), which confirms the Kinect joints chosen by Ozturk et al., (2016) and Valdes et al., (2017) to determine trunk compensation during a reaching task [18, 27]. Consequently, the Kinect joints chosen to be corresponding to CMS20s markers were the right wrist (for right head of the second metacarpal with CMS20s), left wrist (for left head of the second metacarpal with CMS20s) and spine-shoulder (for manubrium with CMS20s).

#### Materials/ setup

To prevent the Kinect from mistakenly identifying furniture as part of the body [28], we omitted the table, used a chair with small arm rests and positioned the target on a narrow stand in front of the participant (see details in Fig. 1). In addition, while the Kinect detects human bodies, it is not able to determine the position of the target. As a consequence, we designed a calibration procedure in which we asked the participants to show where the target was. By asking the participant to reach and stay at the target, with the paretic hand, then with the non-paretic hand, the therapist verified visually that the hand was in place, and recorded for 2 s. The average position of both hands gave the target position. This calibration procedure also included the positioning of the patient in relation to the target.

### Data processing

For the computation of the PANU score, we reasoned that, because the very nature of a reaching task is to reduce the distance to the target [29], we could summarize the relevant information into a 1D space that is, the Euclidean distance from the hand or the trunk to the target. One important consequence of working with distance is to increase the robustness of the assessment procedure in a clinical context: clinician can avoid a long and complex calibration procedure to ensure the orientation of the X, Y and Z axes and the position of the origin. From the Euclidean distance time-series, we first determined the start and the end of each reaching movement. The start position corresponded to the moment when the Euclidean distance from the hand to the target began to decrease (i.e., the hand was no longer resting on the armrest). The end position corresponded to the shortest Euclidean distance from the hand to the target (i.e., the target was reached). Second, we determined the change in Euclidean distance to the target during the reaching movement, for the trunk and for the hand (i.e., ∆Trunk and ∆Hand) so to obtain a value of proximal arm use (i.e., PAU = (∆Hand - ∆Trunk) / ∆Hand) for each reaching movement. From the PAU values, we could compute a PANU score as PANU = MPAU – SPAU, where SPAU is the median PAU value in the spontaneous proximal arm use condition and MPAU is the median PAU value in the maximal proximal arm use condition [3]. In the present study, the PANU scores were calculated for the paretic arm only.

We also extended the comparison of the Kinect and CMS20s towards classical kinematic variables often used to describe upper limb movements after a stroke: Movement Time (MT) and Number of Velocity Peaks (NVP) [30]. The MT was defined as the duration from reach start to reach end. The NVP was defined as the number of peaks in the tangential velocity profile of the hand over the MT.

Finally, we explored one potential added value of the Kinect over the marker-based CMS20s, the fact that it provides the time series of 25 body landmarks that can be used to assess joint movements. Joint angles of interest in relation to the PANU score were measured only with the Kinect system. Since PANU is related to elbow-shoulder non-use [23], we calculated the shoulder and elbow angles using the Kinect data. The elbow flexion/extension was computed from the angle between two vectors in 3D: the forearm (from Elbow to Wrist Kinect joint) and the arm (from Elbow to Shoulder). In order to define the shoulder flexion/extension angle, a frontal plane was created by using 3D coordinates of the spine-mid, spine-shoulder and shoulder right/left. Then, the shoulder flexion/extension was defined as the angle between the vector directed from the shoulder left/right to elbow left/right and the frontal plane [27]. From the shoulder flexion/extension angle and elbow flexion-extension angle, we computed the non-use of shoulder flexion (nuSF) and the non-use of elbow extension (nuEE) by subtracting the angular amplitude in the SPAU condition from the angular amplitude in the MPAU condition.

### Statistical analysis

To quantify the degree to which CMS20s and Kinect measurements are related, we used intra-class correlation coefficient (ICC) and linear regression analysis [31], complemented with Bland and Altman plots with 95% limits of agreement [32]. In these analyses, we pooled the data from the test and retest sessions and compared the values obtained with the Kinect and CMS20s.

To quantify the degree to which the test and retest measurements are related, we again used ICC, linear regression and Bland and Altman plots. In these analyses, we did not pool the data from the Kinect and CMS20s, such that we compared the values obtained over the test and retest sessions separately for the two devices.

In addition, the non-parametric Spearman correlation coefficients were used to assess the relationship between the PANU score, arm impairment (FM-UE score) and arm function (BBT) on the paretic side.

Statistical analyses were performed using R (version 3.5.0). The level of significance for all tests was set at *p* < 0.05.

## Results

### Validity of the Kinect against the CMS20s

**Fig. 2:**
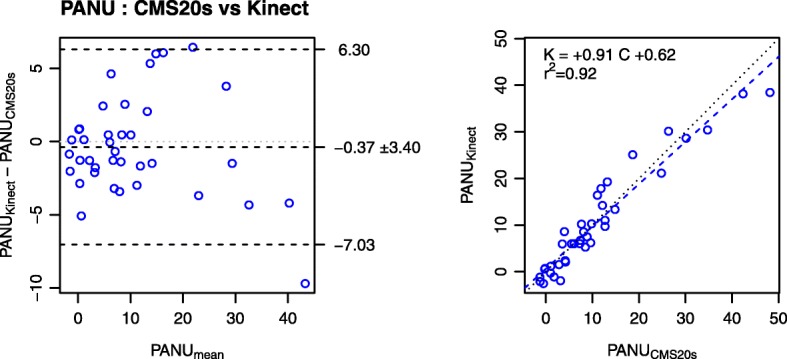
Comparison of PANU scores obtained with the Kinect and CMS20s systems. The left panel presents the Bland and Altman plot and the right panel presents the linear regression plot. PANU scores obtained with the Kinect and CMS20s were strongly correlated, yet with a small underestimate with the Kinect

**Fig. 3:**
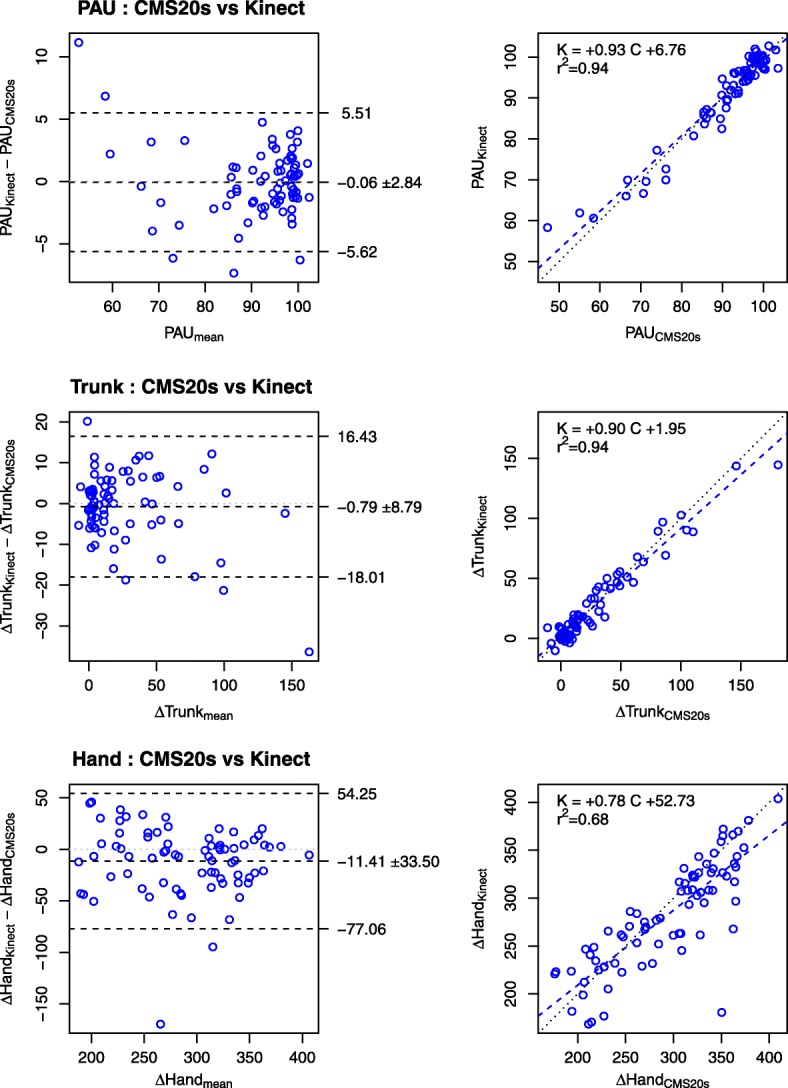
Comparison of PANU components obtained with the Kinect and CMS20s systems. Panels in the first row illustrate proximal arm-use (PAU). Panels in the second row illustrate trunk movement amplitude (∆Trunk). Panels in the third row illustrate reach length (∆Hand). For each row, the left panel represents the Bland and Altman plot and the right panel represents the linear regression plot. The three components are adequately determined by the Kinect, yet with a small underestimate for ∆Hand (11 mm)

**Fig. 4:**
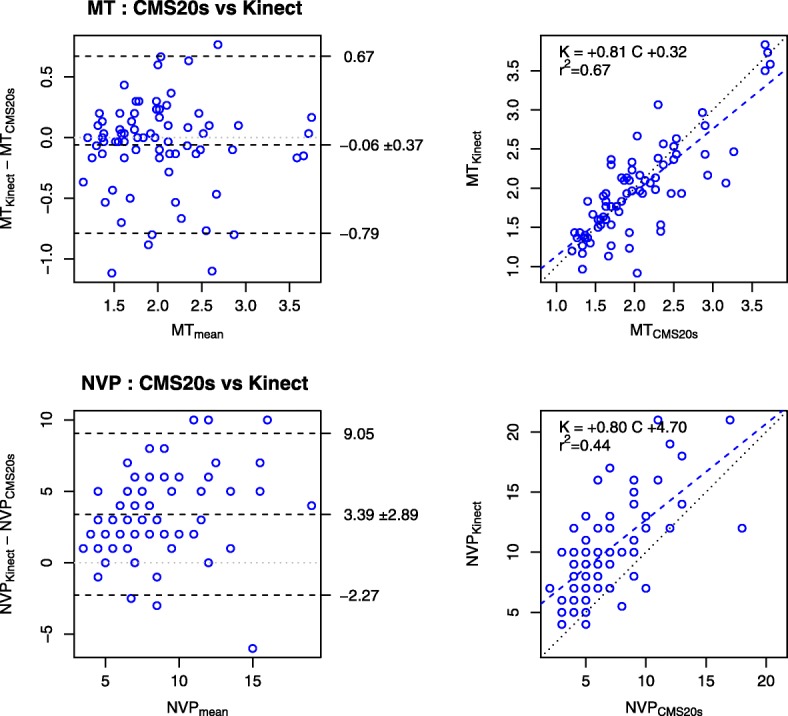
Comparison of movement kinematics obtained with the Kinect and CMS20s. Panels in the first row illustrate the movement time (MT). Panels in the second row illustrate the number of velocity peaks (NVP). For each row, the left panel represents the Bland and Altman plot and the right panel represents the linear regression plot. The movement time is adequately determined by the Kinect, but not the number of velocity peaks

**Fig. 5:**
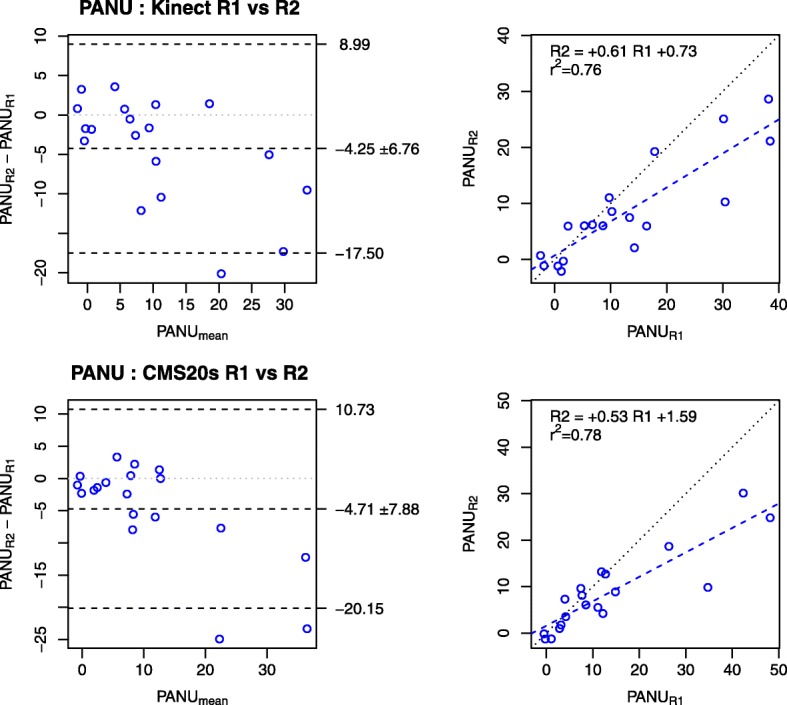
Test-retest of PANU scores with the Kinect and CMS20s. Each panel compares the PANU scores in the test (R1) and retest (R2) sessions. Panels in the first row illustrate repeatability with the Kinect. Panels in the second row illustrate repeatability with the CMS20s. For each row, the left panel represents the Bland and Altman plot and the right panel represents the linear regression plot. The constant bias in the Bland and Altman plots (− 4.25 for Kinect; − 4.71 for CMS20s) indicates that the PANU scores decrease over repetitions, which was accurately determined by the Kinect and the CMS20s

The Bland and Altman analysis is a powerful, yet simple, graphical method to assess the agreement of measurements by 2 methods/sessions: a scatter plot visualises the difference between two measures as a function of the average of the two measures. In a Bland and Altman plot, the central horizontal line indicates the mean of the differences (systematic bias), which is 0 for a perfect agreement. The horizontal lines above and below represent the 95% limits of agreement (average difference ± 1.96 standard deviation of the difference), which tells how far apart measurements by the 2 methods/sessions were more likely to be for most individuals. We complemented the Bland and Altman plots with linear regression analysis, which also provides the equation of the linear regression of one method/session against the other. In Figs. 2, 3, 4, 5 the left panels represent the Bland and Altman plots, and the right panels indicate ICC and regression plots, including the regression equation. Table 1 presents the descriptive statistics of PANU, MT and NVP per recording device.

**Table 1:** Descriptive statistics of the main measures

	MT_[CMS20s]_	NVP_[CMS20s]_	PANU_[CMS20s]_	MT_[Kinect]_	NVP_[Kinect]_	PANU_[Kinect]_
Min	1.20	2.00	−1.27	0.92	4.00	−2.53
Max	3.73	18.00	48.15	3.83	21.00	38.46
Median	1.90	6.00	7.88	1.93	9.00	7.11
IQR	0.74	4.00	9.56	0.77	5.00	14.87
Mean	2.03	6.51	10.92	1.97	9.90	10.55
SD	0.62	3.15	11.75	0.61	3.77	11.16

The three leftmost columns summarize the distributions of MT, NVP and PANU obtained with the CMS20s. The three rightmost columns summarize the distributions of MT, NVP and PANU obtained with the Kinect. The rows in the table indicate the range (Min and Max), the central tendency (Mean and Median) and the variability (SD: standard deviation and IQR: inter quartile range) of the corresponding distribution

The agreement between the Kinect and CMS20s measurements of PANU score, PANU components (PAU, ∆Hand, and ∆Trunk) and kinematic variables (MT and NVP) is assessed using ICC, linear regression and Bland and Altman plots (Figs. 2 and 3).

#### PANU scores

PANU scores obtained from Kinect and CMS20s were strongly correlated with ICC = 0.96. The slope of the linear regression (0.91) indicated that Kinect underestimated PANU (Fig. 2, right panel). The Bland and Altman plots indicated that PANU scores obtained from Kinect were on average 0.37 ± 3.40 shorter than the PANU scores obtained from CMS20s (Fig. 2, left panel).

#### PANU components

##### Pau

For the comparison of PAU, the measurements from the Kinect and CMS20s were strongly correlated with ICC = 0.97. The slope of the linear regression (0.93) indicated that Kinect underestimated PAU (Fig. 3 top panel, right column). The Bland and Altman plots indicated that PAU scores obtained from Kinect were on average 0.06 ± 2.84 shorter than the PAU scores obtained from CMS20s (Fig. 3, top panel, left column).

##### ∆trunk

Change in distance to the target due to trunk movement (∆Trunk) from the Kinect and CMS20s were correlated with ICC = 0.97. The slope of the linear regression (0.90) indicated that Kinect underestimated ∆Trunk (Fig. 3, middle panel, right column). The Bland and Altman plots indicated that ∆Trunk obtained from Kinect was on average 0.79 ± 8.79 mm shorter (Fig. 3, middle panel, left column).

##### ∆hand

Change in distance to the target due to the hand movement (∆Hand) from the Kinect and CMS20s were also highly correlated with an ICC = 0.81. The slope of the linear regression (0.78) indicated that Kinect underestimated ∆Hand (Fig. 3, bottom panel, right column). The Bland and Altman plots indicated that ∆Hand obtained from Kinect was on average 11.41 ± 33.50 mm shorter (Fig. 3, bottom panel, left column).

#### Related kinematic variables

##### Mt

For the movement time (MT), the measurements from the Kinect and CMS20s were correlated with ICC = 0.82. The slope of the linear regression (0.81) indicated that Kinect underestimated MT (Fig. 4, top panel, right column). The Bland and Altman plots indicated that, on average, Kinect measured MT 0.06 s less than CMS20s with a standard deviation of 0.37 s (Fig. 4, top panel, left column).

##### NVP

For the number of velocity peaks (NVP), the measurements from the Kinect and CMS20s were poorly correlated with ICC = 0.34. The large intercept (4.70) did not allow the simple interpretation that the slope of the linear regression (0.80) indicates an underestimate (Fig. 4, bottom panel, right column). The Bland and Altman plots indicated that, on average, Kinect measured NVP with 3.39 peaks more than CMS20s and a standard deviation of 2.89 peaks (Fig. 4, bottom panel, left column).

### Additional kinematic variables that have only been measured by the Kinect

From the time series of 25 body landmarks provided by Kinect data, we examined the non-use of joints determining the PANU score, namely the non-use of shoulders and elbows. We found that PANU was significantly related to nuEE (PANU = + 0.55 nuEE + 7.17 with r^2^ = 0.35, F(1,36) = 19.63, *p* = 0.0001) but not to nuSF (PANU = − 0.27 nuSF + 10.77 with r^2^ = 0.02, F(1,36) = 0.85, *p* = 0.3631).

### Relation of PANU scoring with the clinical assessments

Spearman’s correlation indicated that the PANU score was not significantly linked to the FM-UE (CMS20s: *r* = − 0.32, *p* = 0.24 and Kinect: *r* = − 0.26, *p* = 0.13) and not significantly linked to the BBT (CMS20s: *r* = − 0.10, *p* = 0.57 and Kinect: r = − 0.10, *p* = 0.42). The FM-UE and BBT scores were linked (*r* = 0.60, p = 0.00). The PANU scores measured by the CMS20s and by the Kinect were very strongly linked (*r* = 0.95, p = 0.00) as expected from Fig. 2, right panel.

In addition, among the 11 post-stroke individuals with a high proximal FM-UE score (> 28/42: low impairment), greater than 50% were found with a PANU score > 6.5% (Kinect: 6/11 and CMS20s: 7/11).

### Repeatability of PANU scoring using Kinect and CMS20s

#### Kinect

With the Kinect, PANU scores between the two testing sessions (R1 and R2) were highly correlated (ICC = 0.76) (Fig. 5, top panels). The slope of the linear regression (0.61) indicated that PANU scores at the second session were systematically lower than at the first session. Bland and Altman plots revealed that PANU score at the second session was 4.25 ± 6.76 lower than at the first session.

#### CMS20s

With the CMS20s, PANU scores between the two testing sessions were highly correlated (ICC = 0.72) (Fig. 5, bottom panels). The slope of the linear regression (0.53) indicated that PANU scores at the second session were systematically lower than at the first session. Bland and Altman plots revealed that PANU score at the second session was 4.71 ± 7.88 lower than at the first session.

## Discussion

This study validated that the Kinect sensor can adequately measure proximal arm non-use (PANU score) in patients after a stroke. PANU scores determined by the Kinect system and the reference CMS20s system were highly correlated and repeatable. The components of the PANU score (PAU, ∆Trunk, ∆Hand) were similarly highly correlated. However, other kinematic variables such as movement time (MT) and number of velocity peaks (NVP) obtained from the Kinect were less correlated to that obtained from the CMS20s. Thus, more precise systems than the Kinect are likely necessary to properly detail arm movement organisation. Nevertheless, the marker-less Kinect affords easier access to joint angles than the marker based CMS20s. Furthermore, the Kinect results showed that the majority of patients with lower impairment (FM-UE proximal > 28/42) exhibited proximal arm non-use (PANU score > 6.5%), as already found in our previous paper [3].

### Validation of the PANU scores obtained by Kinect

Our clinical question was whether the Kinect, which is a low cost and marker-less motion capture system, could replace the reference CMS20s system in the assessment of PANU score in patients after a stroke. An ideal model would claim that the measurements obtained by the Kinect and CMS20s would give exactly the same results. So, ideally, this would result in an ICC = 1, linear regression line with slope = 1 and intercept = 0 and coefficient of determination *r* = 1. In the same way, all the differences between paired measurements in the Bland and Altman plots would be equal to 0 (systematic bias) and limits of agreements = 0 as well. Our results indicate that PANU scores determined by the Kinect were similar to those determined by the CMS20s, yet with small discrepancies compared to the ideal expectations.

The origin of the small discrepancies in PANU scores between CMS20s and Kinect are likely twofold: due to measurement inaccuracies of the Kinect and due to the different positioning of the “markers” with the two systems. Inaccuracy in Kinect’s joints is likely due to higher measurement noise (because marker-less tracking is not as stable as marker-based tracking) and also probably due to joint-tracking errors depending on the orientation of body segments relative to the Kinect sensor [33]. For example, it is likely that the Kinect can more easily lose track of the wrist when the hand and elbow are aligned with the sensor (e.g., when moving back after reaching a target).

The different positioning of the “markers” with the two systems can also account for the discrepancies, especially when assessing the trunk movement amplitude (∆Trunk) and the reach length (∆Hand). For ∆Trunk, the Kinect joint was close to the anatomical seventh cervical vertebra while the CMS20s marker was at the body surface on the manubrium, hence with a distance of about 100 mm between the two. However, the error in ΔTrunk measured by the Kinect was about 1 mm on average. Hence, this distance of 100 mm did not impair much the assessment of trunk movements, probably because the trunk moved mainly along the Z axis and remained at a large distance from the centre of the space (i.e., the target). For ∆Hand, the Kinect joint was close to the anatomical centre of the wrist joint while the CMS20s marker was at the body surface, hence about 30 mm to the anatomical centre of the wrist joint. The error in ∆Hand determined by the Kinect was about 11 mm on average, which is small in comparison to the total reach length (280 mm). This might be because the “wrist” was not very well tracked by the Kinect. However, another cause of error was due to (i) the wrist joint, which is close to but not exactly at the position of the CMS20s marker and (ii) the difference generates an error that depends on upper limb orientation. The combination of these causes likely explains the small differences in ∆Hand measurements between the Kinect and CMS20s.

### Test-retest reliability of the Kinect based PANU assessment compared to CMS20s

PANU scores between the two testing sessions were highly correlated with the Kinect (ICC = 0.76) as well as with the CMS20s (ICC = 0.72). Moreover, Bland and Altman plots revealed that PANU scores at the second session were lower than at the first session (4.25 ± 6.76 for the Kinect and 4.71 ± 7.88 for the CMS20s). Therefore, the size of the error in PANU score with the Kinect (− 0.37 ± 3.40) was smaller than the size of the error between two repeated measures on the same person − 4.71 ± 7.88.

The slope of the linear regression (0.61 and 0.53) indicated that the second session PANU scores were systematically lower than the first session with both Kinect and CMS20s. The differences in PANU scores over repetitions might be due to practice effect, since patients may remember to not move their trunk in the spontaneous arm condition of the PANU assessment, as already found with a between day test-retest [3]. This could be explained by patients remembering the task requirements of minimising their trunk movements in the maximal arm use condition of the PANU assessment. However, this provides evidence that specific training reduces trunk compensation that overtime will reduce PANU to acceptable levels.

### Application of Kinect for clinical assessment of PANU

Compared to our previous study [3], the results of this study did not show a significant correlation between FM-UE or BBT and PANU (Kinect and CMS20s). The lower number of patients used in the correlation analysis in the present (*n* = 19) vs previous (*n* = 45) study is a factor for not being able to get a significant relationship between PANU and FM-UE/BBT. However, the non-significant correlation is not fully unexpected, and this is because PANU measures the non-use while FM-UE/BBT measures the deficit/function. On the one hand, PANU measures the non-use as a difference: the difference between spontaneous arm use and maximum arm use. On the other hand, FM-EU/BBT measures the deficit/function as single measure: the ability to make movements on request, which is in a maximal arm use condition. Hence, it is expected that the FM-UE/BBT and the PANU are largely independent measures, because they assess two different domains of arm movement. In addition, the fact that we consistently found in the present and previous [3] study that the majority of patients with lower impairment exhibited proximal arm non-use indicates that these patients do not make full use of their arm movement ability in everyday life. Thus, we think that PANU represents novel information on arm use, which is useful to complement clinical measures of arm impairment and function.

To accomplish arm-reaching movements after damage to the central nervous system, post-stroke individuals generally use an abnormal coupling of the shoulder abduction and elbow flexion generally termed “flexion synergy” [34–36] which is due to a difficulty to isolate degrees of freedom especially in the elbow [23, 37]. Some post-stroke individuals presenting a pathological flexion synergy show a smaller range of motion of the shoulder and elbow joints which is compensated by excessive trunk movements in order to achieve the forward reaching task [6, 38]. Moreover, the amount of compensatory trunk movements classically increases with the severity of the motor deficit [23]. However, two cases of compensatory trunk movements need to be distinguished. On the one hand, if the motor deficit is very severe, trunk compensation might be mandatory to ensure the success of the arm reaching: the patient will not succeed in the same reaching task if trunk movements are restricted. Typically, those patients do not have PANU because they use the same trunk compensation in both the spontaneous and the maximum arm-use conditions. On the other hand, if the motor deficit is not too severe, trunk compensation might not be mandatory: the patient will succeed in the same reaching task whether trunk movements are restricted or not. In the latter case, the amount of trunk compensation to perform the reaching task reveals the *bad-use* of the existing synergies [4, 23]. Here, the compensatory bad-use of the trunk and elbow-shoulder during reaching is diagnosed by the PANU score. This phenomenon can be observed whatever the level of UL recovery.

With the Kinect, the PANU score is assessed with a small error of − 0.37 ± 3.40 compared to the gold standard CMS20s. We think that this small error in PANU score is not clinically important when compared to the typical range of PANU scores of Kinect and CMS20s (− 2.53 to + 48.15, with a median value of 7.58). As a consequence, our results indicate that the Kinect sensor can accurately and reliably determine the PANU score in clinical routine. This result is important because the Kinect has many advantages that are important for practical reasons in clinical routine. The main asset of the Kinect is that it is a marker-less system. Using markers presents several potential problems, including soft tissue artefacts, lost markers, training of therapist to position markers and potentially uncomfortable exposure of areas of the body such as the thorax region that could be a problem for female patients [39]. The other advantages of a Kinect-based system are: 1) easy to set up - no further physical equipment is needed, 2) safe - no additional trip hazards with wires, and 3) inexpensive - the Kinect is a widely available low cost consumer device [12]. These characteristics enable its use in a clinical routine, but also for rehabilitation following-up in conditions where expensive marker-based motion capture methods are difficult to use (e.g., at the patient’s home).

Although the use of the Kinect sensor for virtual rehabilitation of the UL is well demonstrated [12, 40–45], the use of the Kinect for clinical assessment is less well developed. Nevertheless, several studies have already shown that the Kinect sensor can be used for clinical assessments of the UL [13, 46–55], and trunk kinematics [18, 56]. In the present study, the reliability of a Kinect-based assessment of PANU score, which allows differentiating reaching strategies following a stroke, provides a strong case for the routine clinical application of the Kinect for this purpose. Our work reveals that a Kinect based assessment of PANU score is fast, easy to undertake and accurate, hence potentially clinically useful in classifying targeted rehabilitation and follow-up monitoring of UL recovery of post-stroke individuals.

In the present study, in addition to accurately and reliably measuring PANU with the Kinect, we were able to estimate shoulder-elbow angles and derive nuSF and nuEE, with the same setup. In fact, we could measure these angles with CMS20s but this would have required to add more markers on the post-stroke individual. Consequently, we do not have a referent device, which hinders the possibility of a precise assessment of the reliability of the Kinect in the measure of angles (that would be analysed in future work). Our findings with the Kinect suggested that proximal arm non-use (PANU) was mainly due to the inability to extend the elbow joint (nuEE). Yet, Massie et al. found that elbow extension predicts motor impairment and performance after stroke [57]. In our study, the PANU score seems to be more associated with the non-use of elbow extension (nuEE). This additional information can provide useful global UL functional measure to help therapists in focusing the therapy.

To our knowledge, the only clinical scale measuring excessive trunk movements during reach-to-grasp tasks is the Reaching Performance Scale [58]. Unlike the objective PANU assessment, the Reaching Performance Scale focuses on direct assessor’s observation of compensatory movement patterns performed during a reaching task in post-stroke individuals including trunk compensation [58]. Although the Reaching Performance Scale evaluates reach-to-grasp performance (motor impairment) and trunk compensation, it does not provide an indication of arm non-use [2, 59–61]. Recently, Valdés et al. measured changes in anterior trunk displacement using the Kinect in a bimanual reaching task [18]. The PANU method also evaluates trunk displacement using the ∆Trunk variable. Yet, because PANU compares ∆Trunk when reaching in a spontaneous condition to ∆Trunk in a maximal proximal arm use condition, the PANU assessment method provides new information on maladaptive trunk compensation (i.e., compensations that are not mandatory to succeed at the task).

## Limitations

This study faces several limitations. First, the sample size used in the clinical evaluation was small. Second, the comparison of the Kinect system was done on only one reference (CMS20s), but we could also compare to others systems such as optoelectronic devices. Moreover, the generalization of these results has to be confirmed since the participants in our sample do not reflect all types of deficits after a stroke. Finally, the chosen task is a horizontal forward reaching task and other tasks may yield different results.

## Future work

Motion capture systems such as the Kinect are mostly conducted in upper limb stroke rehabilitation to increase the motivation during training and may assist improvement on one or more International Classification of Functioning, Disability and Health (ICF) levels.

Future work could focus on developing a video game that reduces the PANU. We believe that PANU score is important for rehabilitation programs that aim to reduce the arm non-use in the paretic arm itself and to promote previous movement patterns. Future research might test the hypothesis that post-stroke individuals with a high PANU score might improve with rehabilitation focused on the use of their paretic shoulder flexion and elbow extension.

Therefore, the PANU score could be implemented in virtual/robotic rehabilitation to monitor arm-use over time. Ideally, the new game would be able to measure the proximal arm non-use (assessment part). The game would also be able to meaningfully use arm movements and PANU score as inputs and use incentives and disincentives to reduce PANU (treatment part). The selected training programmes would incorporate different forms of forced-use feedback of the paretic arm provided by the virtual or robotic devices [18] and games to promote the use of the paretic arm [62].

In addition, motion-based games show promise for motivating patients to perform stroke rehabilitation exercises at home by themselves [12]. In the near future and with further validation, Kinect based video games may prove useful as a home-based assessment tool (e.g., monitoring recovery of proximal arm-use [3], monitoring changes in motor synergy patterns over time [63]) and as home-based self-rehabilitation tool [64].

## Conclusion

Our goal was to develop a valid and reliable methodology, easy to administer, time-efficient, and cost-effective to capture PANU in individuals post-stroke. The present study showed that the low cost and marker-less Kinect based motion capture system could accomplish these goals adequately. PANU assessment using the Kinect motion capture sensor could be recommended for rehabilitation (both in hospitals and private practices), such that we envision that PANU could help therapists to 1) classify patients as a function of their unused motor reserve, so to guide patients towards specific arm-use rehabilitation, 2) monitor recovery over time, to assess the effectiveness of interventions.

## Abbreviations

BBT: Box and Block Test
FM-UE: Fugl-Meyer Assessment of the upper extremity
ICC: Intraclass Correlation Coefficient
MT: Movement Time
NVP: Number Velocity Peak
PANU: Proximal Arm Non-Use
PAU: Proximal Arm Use
SD: Standard Deviation
UL: Upper Limb
